# Cortical lesions causing loss of consciousness are anticorrelated with the dorsal brainstem

**DOI:** 10.1002/hbm.24892

**Published:** 2020-01-06

**Authors:** Samuel B. Snider, Joey Hsu, R. Ryan Darby, Danielle Cooke, David Fischer, Alexander L. Cohen, Jordan H. Grafman, Michael D. Fox

**Affiliations:** ^1^ Departments of Neurology, Massachusetts General Hospital and Brigham and Women's Hospital Harvard Medical School Boston Massachusetts; ^2^ Berenson‐Allen Center for Noninvasive Brain Stimulation and Division of Cognitive Neurology, Department of Neurology Beth Israel Deaconess Medical Center Boston Massachusetts; ^3^ Department of Neurology Vanderbilt University Medical Center Nashville Tennessee; ^4^ Department of Neurology Boston Children's Hospital, Harvard Medical School Boston Massachusetts; ^5^ Rehabilitation Institute of Chicago Chicago Illinois; ^6^ Department of Physical Medicine and Rehabilitation, Neurology, Cognitive Neurology and Alzheimer's Center, Department of Psychiatry, Feinberg School of Medicine and Department of Psychology, Weinberg College of Arts and Sciences Northwestern University Chicago Illinois; ^7^ Department of Neurology, Massachusetts General Hospital Harvard Medical School Boston Massachusetts; ^8^ Athinoula A. Martinos Center for Biomedical Imaging Charlestown Massachusetts

**Keywords:** brainstem, coma, functional connectivity, reticular activating system, traumatic brain injury

## Abstract

Brain lesions can provide unique insight into the neuroanatomical substrate of human consciousness. For example, brainstem lesions causing coma map to a specific region of the tegmentum. Whether specific lesion locations outside the brainstem are associated with loss of consciousness (LOC) remains unclear. Here, we investigate the topography of cortical lesions causing prolonged LOC (*N* = 16), transient LOC (*N* = 91), or no LOC (*N* = 64). Using standard voxel lesion symptom mapping, no focus of brain damage was associated with LOC. Next, we computed the network of brain regions functionally connected to each lesion location using a large normative connectome dataset (*N* = 1,000). This technique, termed lesion network mapping, can test whether lesions causing LOC map to a connected brain circuit rather than one brain region. Connectivity between cortical lesion locations and an a priori coma‐specific region of brainstem tegmentum was an independent predictor of LOC (*B* = 1.2, *p* = .004). Connectivity to the dorsal brainstem was the only predictor of LOC in a whole‐brain voxel‐wise analysis. This relationship was driven by anticorrelation (negative correlation) between lesion locations and the dorsal brainstem. The map of regions anticorrelated to the dorsal brainstem thus defines a distributed brain circuit that, when damaged, is most likely to cause LOC. This circuit showed a slight posterior predominance and had peaks in the bilateral claustrum. Our results suggest that cortical lesions causing LOC map to a connected brain circuit, linking cortical lesions that disrupt consciousness to brainstem sites that maintain arousal.

## 1 INTRODUCTION

Consciousness depends on ascending projections from the dorsal brainstem that form what is commonly known as the ascending reticular activating system (Magoun, 1952). Lesion mapping studies in patients with or without coma have refined this topography, identifying a coma‐specific region of the brainstem tegmentum (Fischer et al., 2016; Parvizi & Damasio, 2003).

Outside the dorsal brainstem, the neuroanatomical substrate of human consciousness remains unclear (Boly et al., 2017; Dehaene & Changeux, 2011; Koch, Massimini, Boly, & Tononi, 2016). Functional neuroimaging studies of normal subjects with varying levels of conscious awareness (Lumer, Friston, & Rees, 1998; Siclari et al., 2017), patients with disorders of consciousness (Demertzi et al., 2015; Threlkeld et al., 2018; Vanhaudenhuyse et al., 2010; Wu et al., 2015), and patients under anesthesia (Greicius et al., 2008; Qiu, Scheinost, Ramani, & Constable, 2017) have identified several candidate brain regions and brain networks. However, the regions and networks are inconsistent across studies, fueling debates such as whether cortical regions supporting consciousness are primarily in the front or back of the brain (Boly et al., 2017; Koch et al., 2016; Odegaard, Knight, & Lau, 2017). One point of agreement in these debates is the importance of studying patients with disrupted consciousness following focal brain lesions (Boly et al., 2017; Dehaene & Changeux, 2011). Unlike functional neuroimaging, lesion studies enable causal links between symptoms and neuroanatomy (Adolphs, 2016; Fox, 2018; Rorden & Karnath, 2004).

Cohorts of patients with penetrating head trauma are well suited to help answer this question (Gressot et al., 2014; Muehlschlegel et al., 2016; Salazar et al., 1986). Lesions from penetrating head trauma are predominantly cortical yet 40–60% result in loss of consciousness (LOC) (Salazar et al., 1986). LOC in these patients is associated with larger lesions (Chau, Salazar, Krueger, Cristofori, & Grafman, 2015), left‐hemispheric lesions (Salazar et al., 1986), and possibly lesions to the claustrum (Chau et al., 2015). However, smaller lesions not meeting these criteria can still cause LOC (Chau et al., 2015; Salazar et al., 1986) and a whole‐brain voxel‐based search for lesion locations associated with LOC has not yet been conducted.

It is possible that cortical lesions causing LOC may not map to a single brain region, but to a connected brain network. Lesions causing a variety of different neuropsychiatric symptoms have recently been mapped to brain networks using a technique termed lesion network mapping (Boes et al., 2015; Darby, Laganiere, Pascual‐Leone, Prasad, & Fox, 2017; Fasano, Laganiere, Lam, & Fox, 2017; Fischer et al., 2016; Fox, 2018; Laganiere, Boes, & Fox, 2016). With this technique, a large normative MRI database of resting‐state functional connectivity (rs‐fcMRI) is used to identify brain regions functionally connected to each lesion location. Functional connectivity differences between lesions causing or not causing a given symptom can then be identified.

In this study, we examined brain lesions from patients with penetrating head trauma in the Vietnam Head Injury Study (Salazar et al., 1986). First, we used conventional voxel‐lesion‐symptom mapping to test for associations between lesion location and LOC. Next, we used lesion network mapping to test for associations between lesion connectivity and LOC. We focused on connectivity between lesion locations and the dorsal brainstem given the established role of this region in supporting human consciousness (Fischer et al., 2016; Fuller, Sherman, Pedersen, Saper, & Lu, 2011; Hindman et al., 2018; Parvizi & Damasio, 2003). We hypothesized that cortical lesions producing LOC could be defined by their functional connectivity to a recently described coma‐specific area of the brainstem tegmentum (Fischer et al., 2016). Finally, we used voxel‐wise statistics to map the exact location of the brainstem region most associated with LOC‐producing lesions, and the functional network associated with this region.

## 2 METHODS

See Supplementary Material for additional methodologic details.

### 2.1 Lesion mapping

Details of this cohort have been previously published (Chau et al., 2015; Raymont, Salazar, Krueger, & Grafman, 2011; Salazar et al., 1986). Briefly, patients were selected from a registry of *N* = 1,131 Vietnam War veterans (all men) who survived a penetrating head injury during combat, and for whom detailed medical records beginning as early as 10 minutes postinjury were available. A total of *N* = 520 of these initial registry patients (Raymont et al., 2008) responded to follow‐up mail correspondence and participated in longitudinal clinical follow‐up as part of the Vietnam Head Injury Study (Salazar et al., 1986). We studied a subset of 171 of these patients for which data existed about LOC at the time of the injury (Salazar et al., 1986). Patient demographics are listed in Table S1. Investigators abstracted clinical information about LOC by analyzing intake assessments conducted by neurosurgeons and other medical personnel working at MASH units in Vietnam (Salazar et al., 1986). Patients were determined to have had LOC if they appeared unconscious and were unresponsive to verbal commands. Patients were seen in these units within a median of 3 hours from their injury. In most patients, this was the first well‐documented report of their level of consciousness. For some patients, there was earlier information provided to the treating clinicians by witnesses or other military personnel. All surviving patients underwent computed tomography (CT) brain scans during follow‐up, 35 years after the initial injury. CT images were acquired on a GE Electric Medical Systems Light Speed Plus CT scanner at the Bethesda Naval Hospital in Maryland. Voxels were 0.4 × 0.4 mm^2^, with 2.5 mm slice thickness and a slice interval of 1 mm. Manual tracing of encephalomalacic lesions on CT scans was conducted by a trained neuropsychiatrist and then reviewed by a researcher (J.H.G.) blind to the clinical outcomes. A consensus judgment defined the final outline of the lesion or lesions. The resulting volumes were spatially normalized to Montreal Neurological Institute (MNI) space using automated image registration and a 12‐parameter affine fit (Chau et al., 2015). All subsequent analyses including voxel‐wise analyses were performed using custom scripts in MATLAB (MathWorks, Natick, MA) and R (https://www.r-project.org/), and images were visualized using FMRIB Software Library (FMRIB Analysis Group, Oxford University, UK).

We defined LOC ordinally, with groups corresponding to no, brief (<1 day), or prolonged (>1 day) LOC, as in prior work (Chau et al., 2015; Salazar et al., 1986). We used ordinal logistic regression to test different brain regions and functional connections for correlation with LOC. We confirmed the proportional odds assumption in each case using a likelihood ratio statistic (Supplementary Methods). This analysis is slightly different than prior work using this dataset, which treated frequency of LOC in each group and duration of LOC as separate variables (Chau et al., 2015). Our current approach was chosen to simplify the analysis to a single outcome variable and maximize statistical power.

### 2.2 Lesion symptom mapping

We performed voxel‐wise regressions on parenchymal voxels lesioned in at least five veterans, correcting for lesion size. *p *Values were adjusted for multiple comparisons using Benjamini–Hochberg false discovery rate (FDR) correction (Benjamini & Yosef, 1995). To ensure that negative results were not due to details of this regression, we repeated the analysis multiple times, variably restricting the analysis to voxels lesioned in 5 versus 10 veterans and including or excluding lesion size as a covariate.

### 2.3 Lesion network mapping

Full methodological details regarding the acquisition and preprocessing of the normative, 1,000‐subject rs‐fcMRI dataset have been published previously (Yeo et al., 2011). Preprocessing included global signal regression and spatial smoothing at 6 mm full width at half maximum. Global signal regression is a technique that increases neuroanatomical specificity by non‐specific sources of variance in the blood oxygen level dependent (BOLD) signal (Fox et al., 2005; Fox, Zhang, Snyder, & Raichle, 2009; Murphy & Fox, 2017). In the region of interest (ROI) to ROI analyses, we generated Fisher Z‐transformed Pearson *R* values representing connectivity between each lesion location and the coma‐specific area. For voxel‐wise analyses, we calculated the Fisher Z‐transformed Pearson *R* values between each lesion location and every other voxel within a brain mask in MNI space.

Using ordinal logistic regression, we tested whether LOC was associated with the ROI to ROI connectivity between lesion location and the coma‐specific area (Fischer et al., 2016), with and without correction for other significant lesion characteristics including lesion size and hemispheric involvement. To investigate the spatial topography of significant findings, we repeated this analysis on a voxel‐wise basis, regressing each voxel's set of Fisher Z connectivity values with each lesion against the set of subjects' LOC values. We initially generated voxel‐wise generalized linear models using FMRIB Software Library's (FSL) PALM tool, which employs permutation testing and threshold free cluster enhancement (Smith & Nichols, 2009) to control for multiple comparisons across brain voxels (Family Wise Error rate (FWE) *p* < .05). We restricted the analysis to a pons and midbrain mask generated using an automated freesurfer segmentation of the MNI 152 T1 template brain and compared both univariate and multivariate (including lesion volume and hemispheric involvement) models. We then repeated this analysis within a whole brain mask using voxel‐wise ordinal logistic regressions, variably including lesion size and hemispheric involvement as covariates.

To assess the effect of global signal regression on our results, we repeated our ROI to ROI analysis using a separate preprocessing pipeline on the first 100 subjects of the Brain Genomics Superstruct Project (Holmes et al., 2015), a subset of the 1,000‐subject connectome. Resting‐state data in this subset was processed using the aCompCor strategy as implemented in the Conn Toolbox (http://www.nitrc.org/projects/conn) (Whitfield‐Gabrieli & Nieto‐Castanon, 2012). In brief, subject‐specific anatomical noise ROIs were obtained by segmenting the white matter and CSF. Principal components analysis was performed on the signals in the white matter and CSF noise ROIs, and the first five components were used as nuisance regressors in a general linear model. Other nuisance regressors included the six motion parameters and their derivatives and time series representing outlier artifactual frames as estimated by artifact rejection software (http://www.nitrc.org/projects/artifact_detect/) integrated in Conn. All settings for preprocessing and regression were kept as default in the Conn Toolbox.

## 3 RESULTS

### 3.1 Lesion mapping

**Figure 1:**
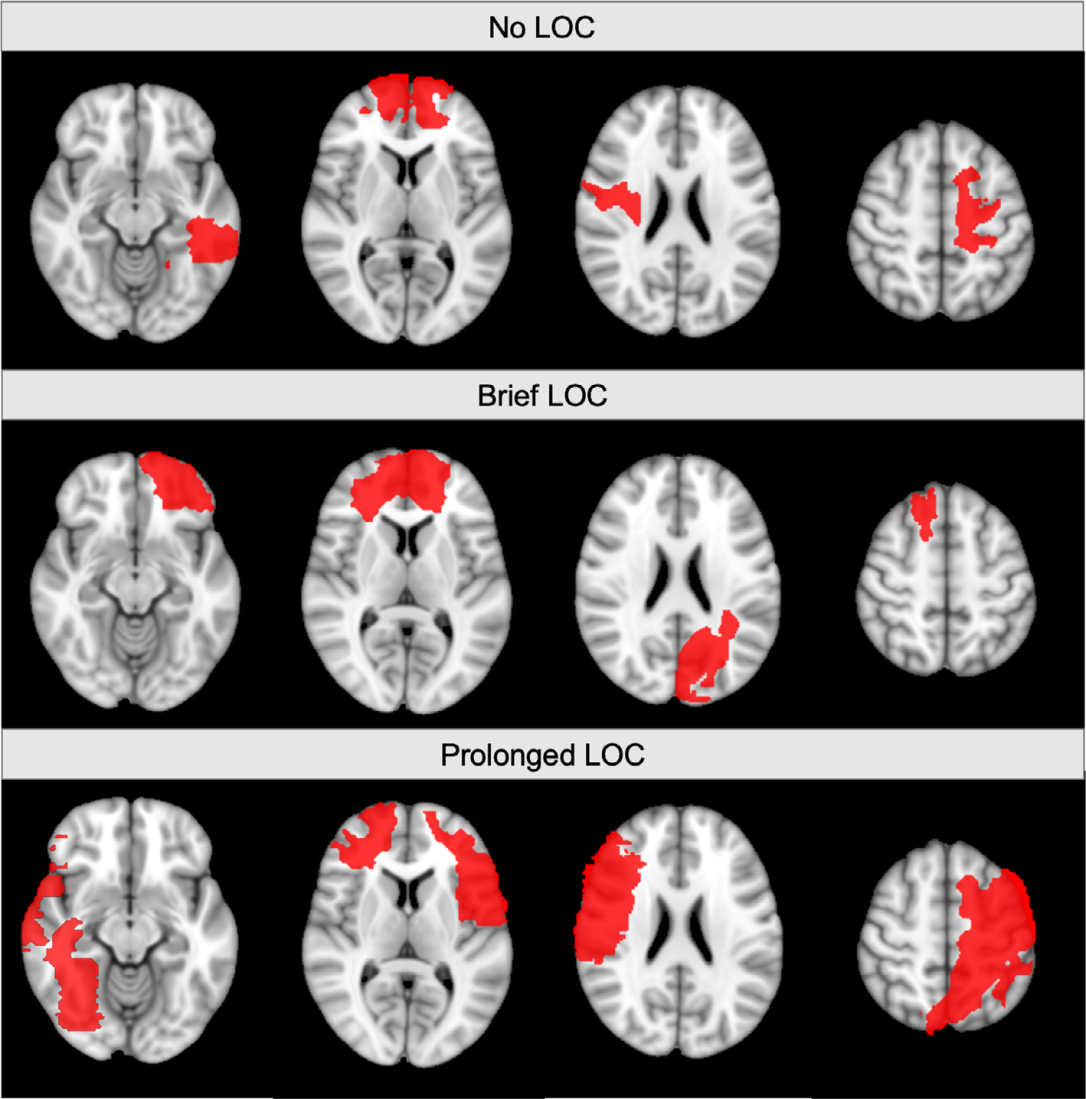
**Representative lesion locations and their association with loss of consciousness**. Single axial slices of four representative lesions in patients with no loss of consciousness (LOC), brief LOC, or prolonged LOC are shown, demonstrating heterogeneity in lesion location

**Figure 2:**
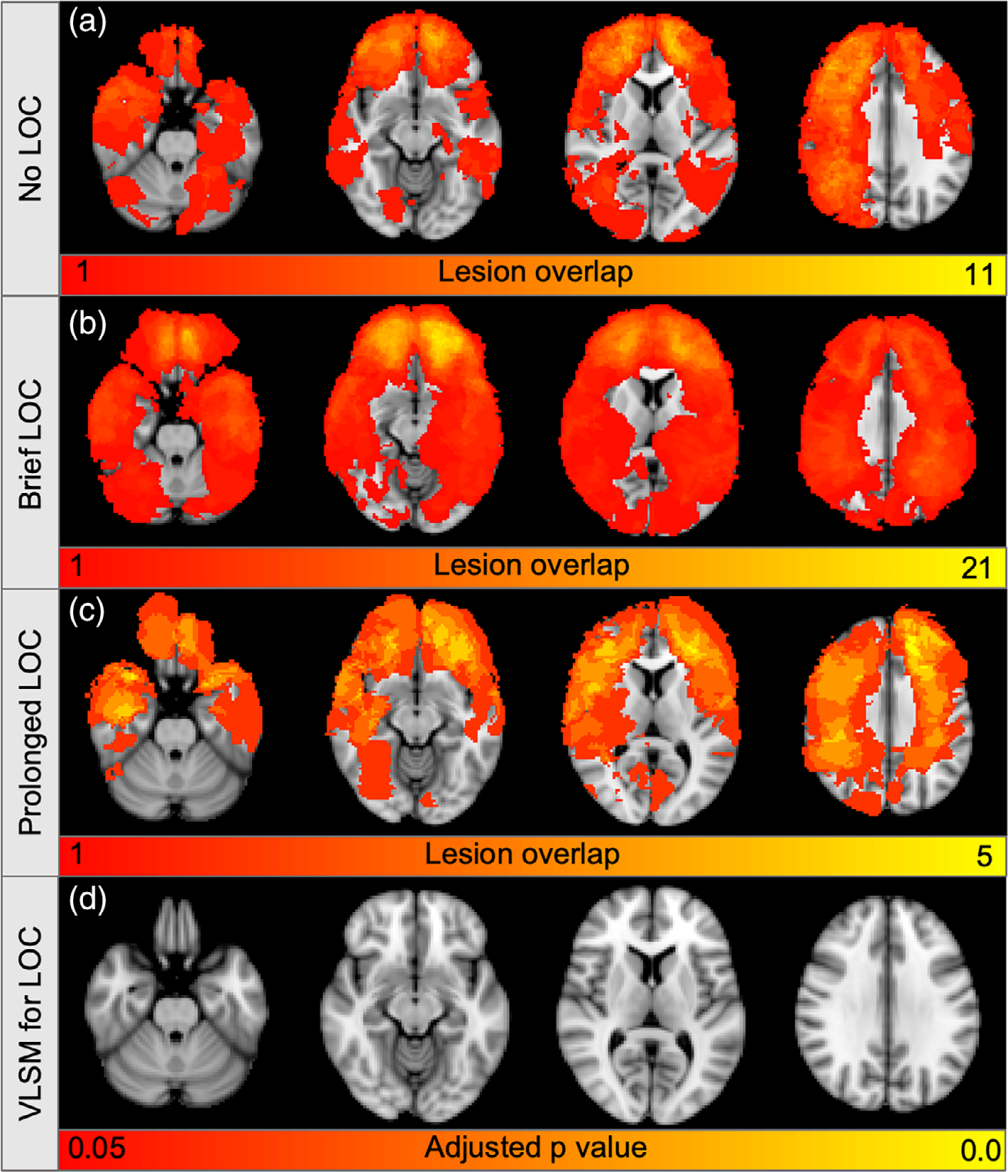
**Lesion locations are not significantly associated with loss of consciousness**. Color indicates the total number of lesions at each voxel in patients with no loss of consciousness (LOC) (*N* = 64, a), brief LOC (*N* = 91, b), or prolonged LOC (*N* = 16, c), with yellow set to the maximum lesion overlap in each patient subgroup. (d) Among voxels lesioned in at least five patients, voxel lesion symptom mapping (VLSM) identified no voxels significantly associated with LOC (all FDR‐adjusted *p* > .05)

Lesions causing no LOC (*N* = 64), brief LOC (*N* = 91), or prolonged LOC (*N* = 16) occurred in a variety of different brain locations (Figure 1). Consistent with prior work, larger lesions, and left hemispheric lesions were independently associated with LOC (all *p* < .0005, Tables S2 and S3). Most lesions (151/171) were limited to the cortex and underlying white matter. Deeper lesions (*N* = 20) were not more likely than superficial lesions to cause LOC (*B* = 1.1, *p* = .8), but no deep lesions intersected the dorsal brainstem. Voxel‐lesion symptom mapping failed to identify any voxels significantly associated with LOC despite trying several variations of this analysis (Section 2) and including or excluding lesion size as a covariate (Figure 2).

### 3.2 Lesion network mapping

**Figure 3:**
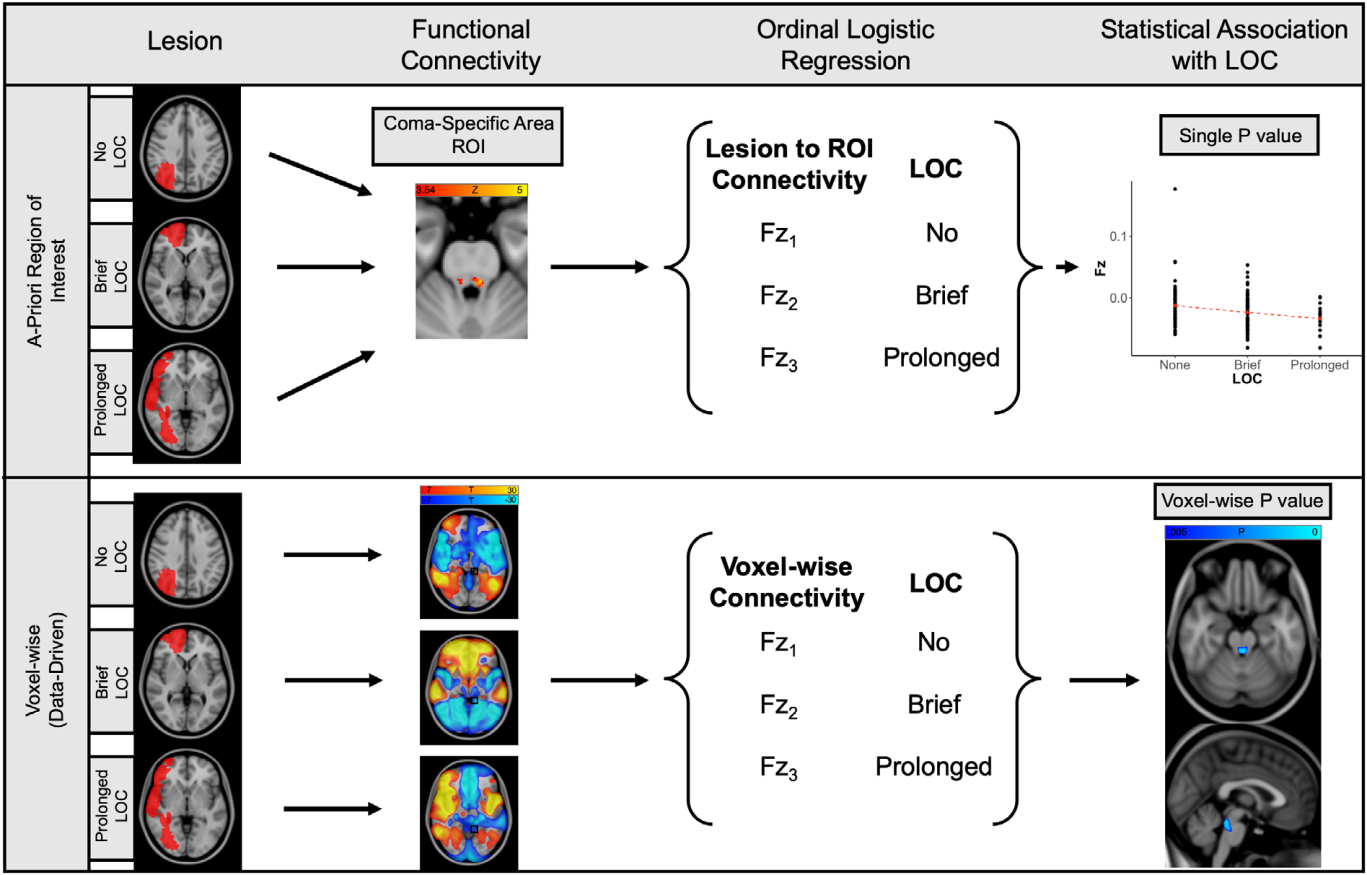
**Schematic depiction of lesion network association mapping**. In the top row, the functional connectivity between each lesion location and a pre‐specified region of interest (ROI) is measured in a normative dataset of *N* = 1,000 rs‐fMRI scans. The resulting distribution of Fisher Z‐transformed Pearson *R* values is regressed against each subject's ordinally defined duration of unconsciousness. In the bottom row, the functional connectivity between each lesion location and every other voxel in Montreal Neurological Institute (MNI) space is measured in the normative dataset. The set of Fisher Z values at each voxel within a mask of interest is regressed against each subject's ordinally defined duration of unconsciousness. The multiple‐comparison adjusted (threshold free cluster enhancement) *p* values for the beta weights of each voxel are then plotted in MNI space

**Figure 4:**
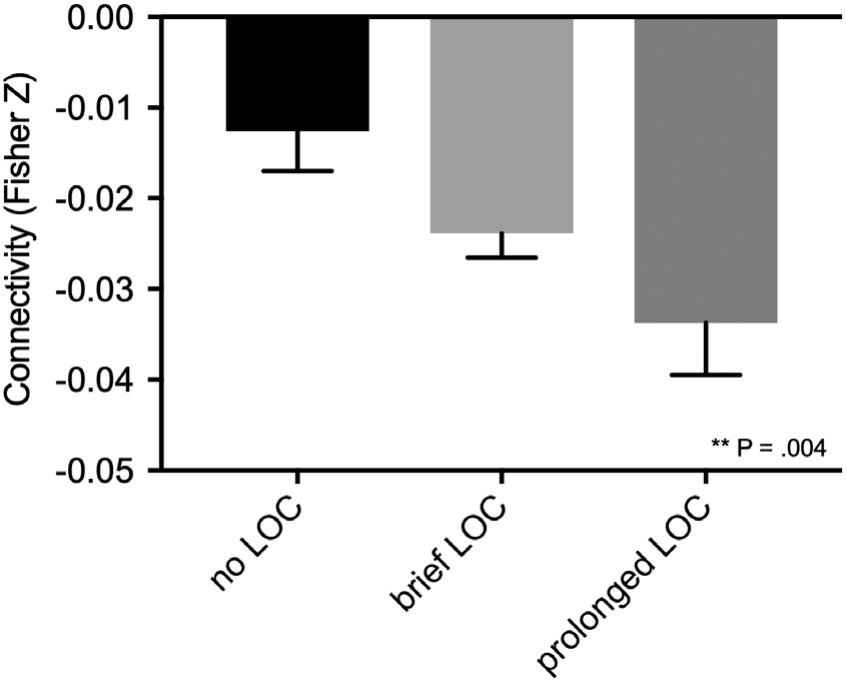
**Connectivity between lesion locations and the coma‐specific region in the dorsal brainstem is significantly associated with loss of consciousness**. Mean and *standard error* of the functional connectivity (Fisher‐transformed Pearson's *R*) between lesion location and a region of interest specific to coma‐causing brainstem lesions. Lesion locations associated with loss of consciousness (LOC) are more negatively correlated (anticorrelated) with this brainstem region. *p* Value reflects significance in a univariate ordinal logistic regression

We next used a large database of rs‐fcMRI from healthy participants (*N* = 1,000) to identify brain areas functionally connected to each lesion location (Yeo et al., 2011), as depicted schematically in Figure 3. Functional connectivity between lesion location and our previously defined coma‐specific area of the brainstem tegmentum (Fischer et al., 2016) was significantly associated with LOC (*B* = 1.2 [per −.01 change in *F*z], *p* = .004, Figure 4). This connectivity remained a significant and independent predictor when lesion volume (*B* = 1.2, *p* = .01) or lesion volume and hemisphere (*B* = 1.2, *p* = .002) were included as covariates. Connectivity to this brainstem ROI also remained a significant predictor after excluding the 20 lesions with subcortical involvement (*B* = 1.2, *p* = .02).

Surprisingly, this relationship was driven by negative functional connectivity (anticorrelation) to this brainstem region, as lesion locations more anticorrelated to the dorsal brainstem were more likely to cause LOC (Figure 4). This result was not an artifact of our resting‐state functional connectivity processing methodology, as it persisted when we repeated our analysis using connectome data (*N* = 100) processed without global signal regression (B = 1.1, *p* = .03).

**Figure 5:**
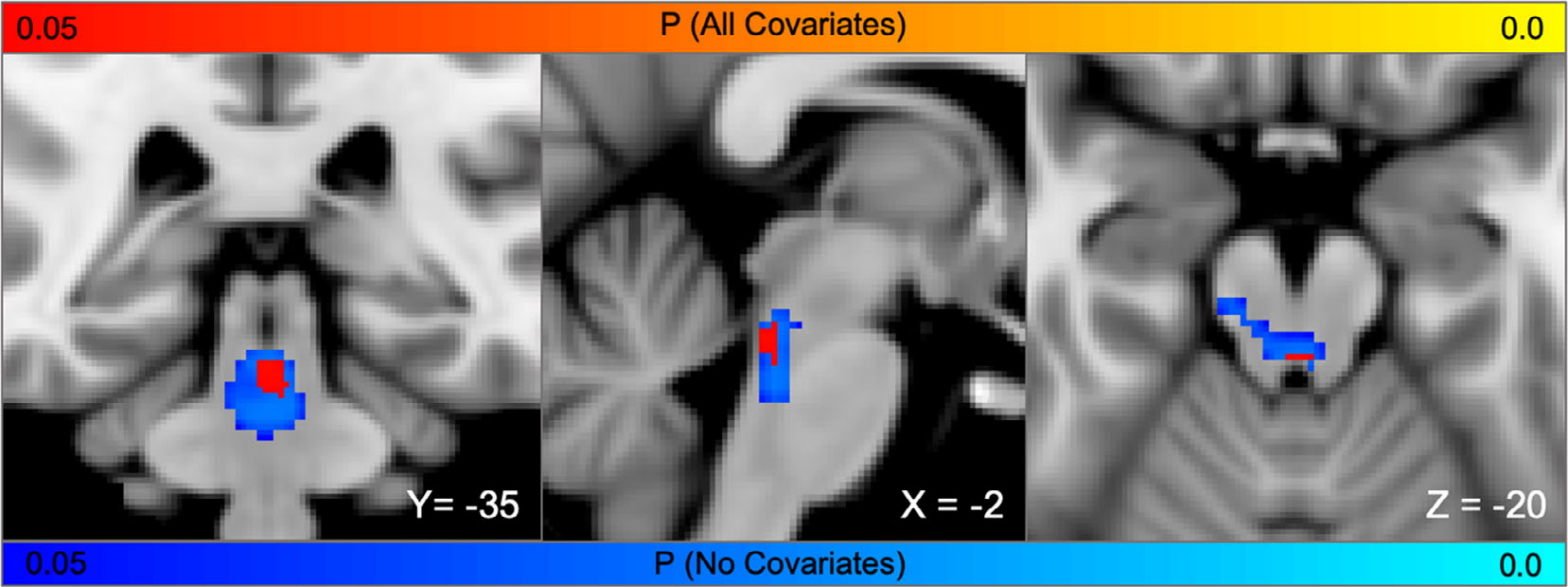
**Topography of brainstem connections most associated with loss of consciousness**. Brainstem voxels whose connectivity with lesion location is significantly associated with loss of consciousness (FWE *p* < .05). Results are similar for a univariate analysis (blue‐light blue) and a multivariate analysis with lesion volume and hemisphere included (red‐yellow)

**Figure 6:**
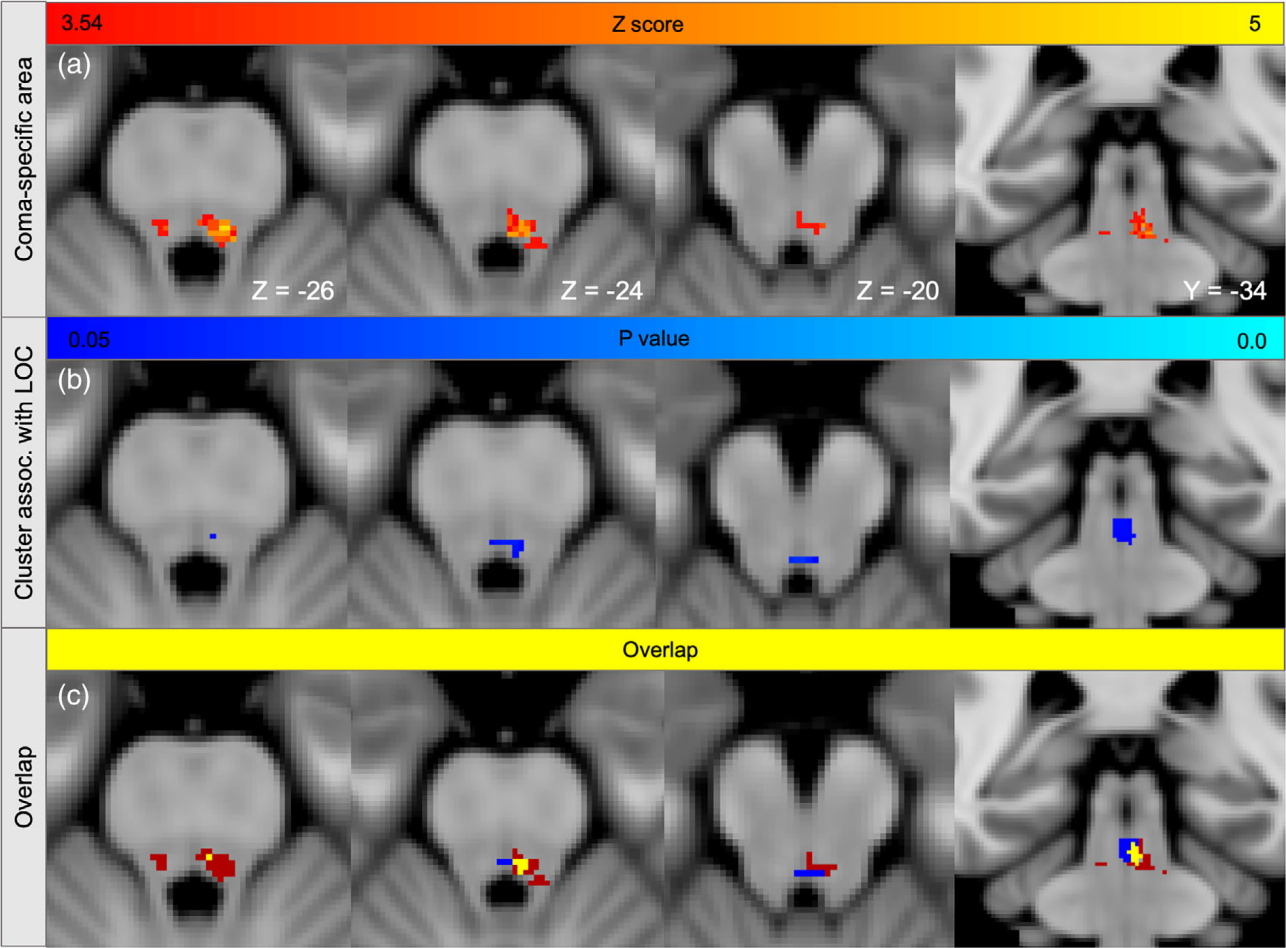
**Brainstem connections associated with loss of consciousness overlap the coma‐specific region**. (a) Lesioned voxels significantly associated with brainstem coma (*Z* > 3.54) from prior work are displayed on three axial slices and one coronal slice in MNI space. (b) Voxels whose connectivity to cortical lesion locations was significantly associated with loss of consciousness (FWE‐significant *p* values in all regressions from Figure 5). (c) Overlap between Panels a and b (yellow)

**Figure 7:**
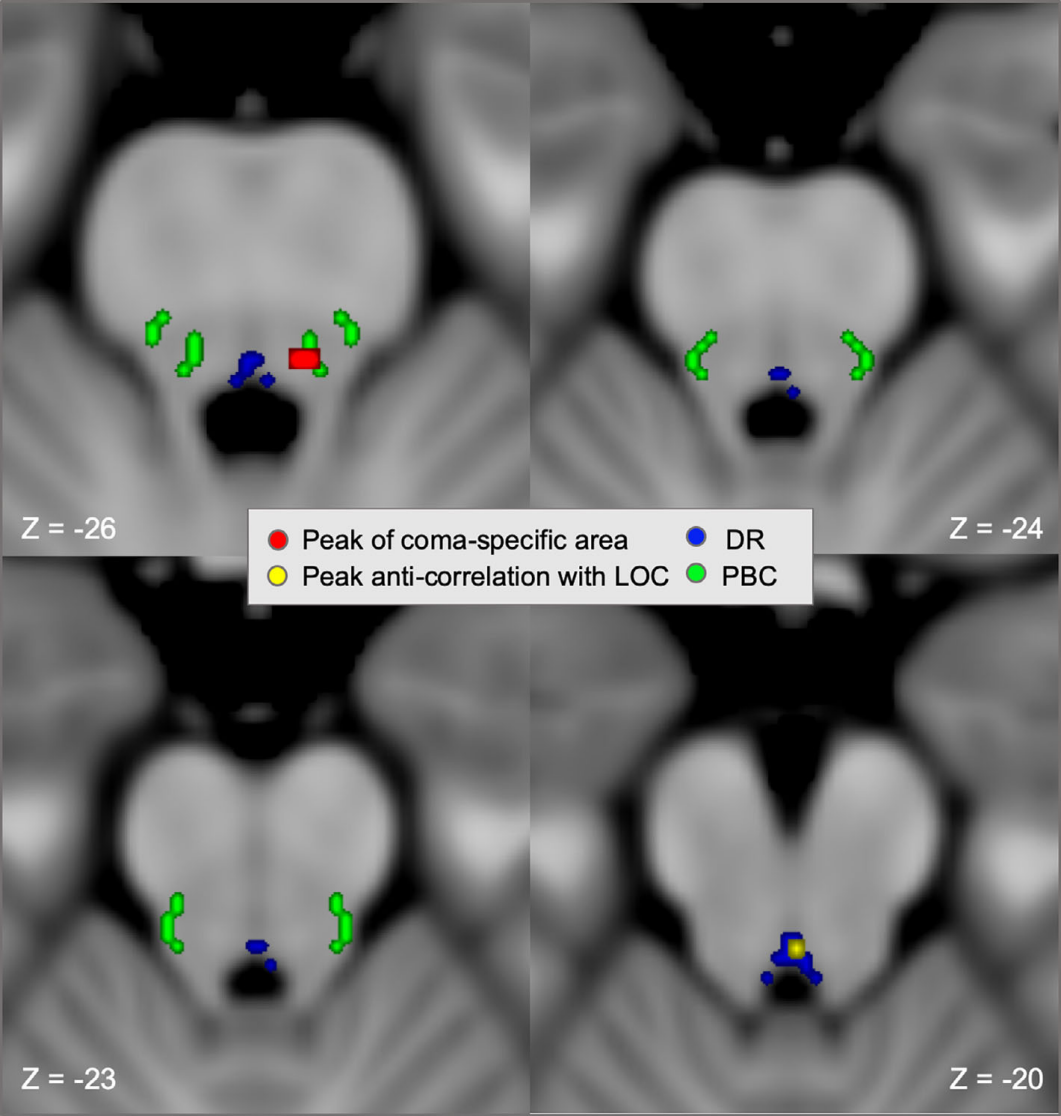
**Coma specific area and peak brainstem cluster overlap distinct arousal nuclei**. Axial slices of brainstem showing the peaks of the coma‐specific area and the current brainstem cluster are overlaid on an MNI atlas of brainstem arousal nuclei (https://www.nmr.mgh.harvard.edu/resources/aan-atlas). The peak of the coma‐specific brainstem region (MNI ‐5, ‐35, ‐26, red, peak voxels from Figure 6a) overlaps the parabrachial complex (green), while the peak of the brainstem cluster functionally connected to loss of consciousness (LOC)‐producing cortical lesions (MNI 0, ‐34, ‐20, yellow, peak voxel from Figure 6b) overlaps the dorsal raphe (blue)

To identify the precise location in the brainstem most associated with LOC, we repeated our connectivity analysis on a voxel‐wise basis within an anatomically defined brainstem mask. We found a significant cluster in the brainstem tegmentum that showed similar topography independent of which coregressors were included (Figure 5). This cluster overlapped our a priori coma‐specific ROI (Fischer et al., 2016) in the dorsal brainstem tegmentum (Figure 6). However, the peak voxel within this cluster was actually rostral and medial to our coma‐specific area, aligning better with the location of the dorsal raphe (Edlow et al., 2012) (Figure 7).

To screen for associations with LOC outside the brainstem, we performed a whole‐brain voxel‐wise search at a relaxed statistical threshold (uncorrected *p* < .001). The whole‐brain peak was in the dorsal brainstem, and no gray‐matter clusters outside the brainstem were identified (Figure S1). Results were again similar independent of the included covariates or the processing methodology (Figure S2).

**Figure 8:**
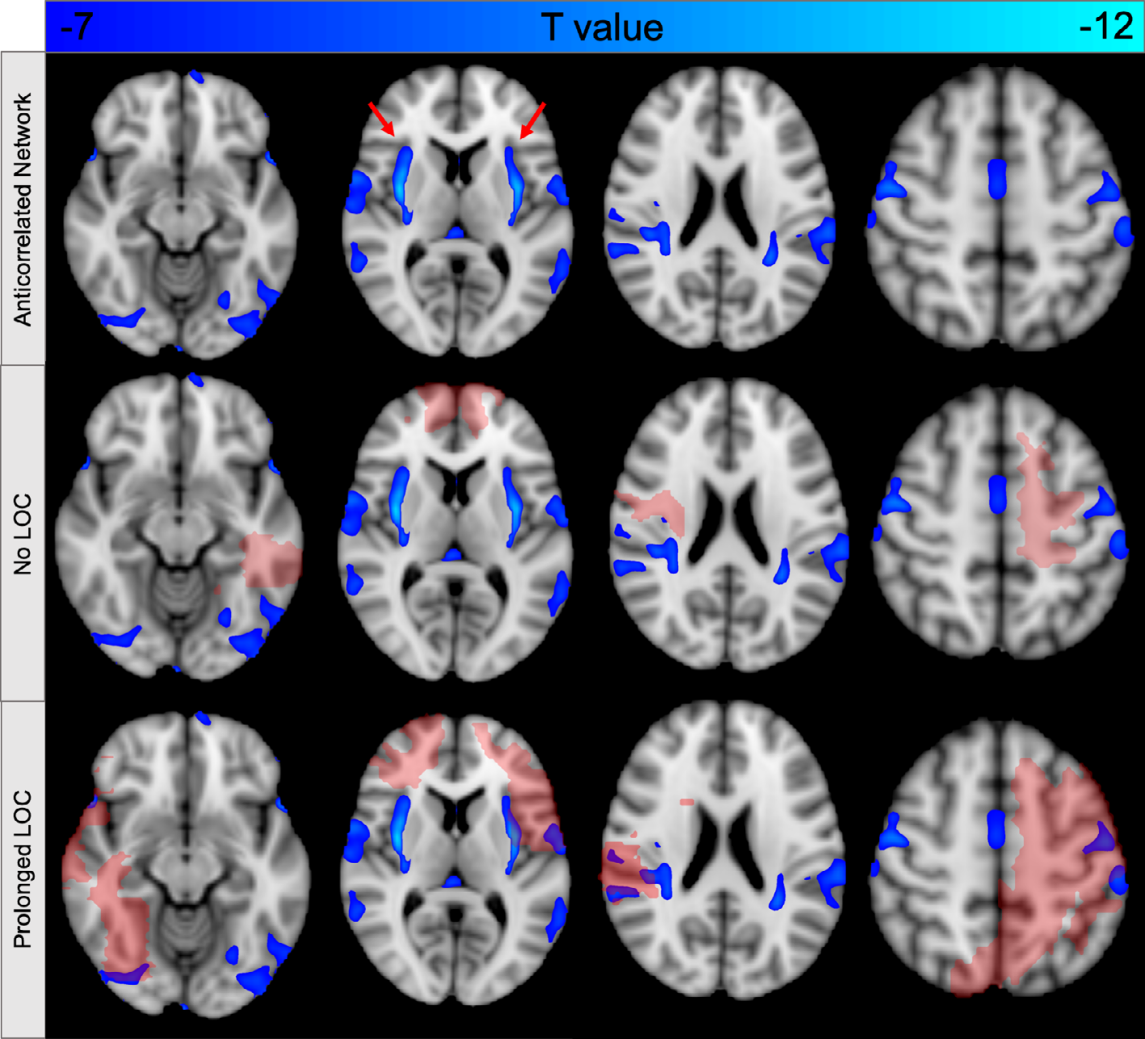
**Functional connectivity with the dorsal brainstem defines a brain network that encompasses lesion locations associated with loss of consciousness (LOC)**. By definition, negative functional connectivity with our dorsal brainstem cluster (Figure 6b) defines a distributed brain network (top row) that avoids lesion locations with preserved consciousness (middle row) and intersects lesion locations associated with LOC (bottom row). The peak of this network is in the bilateral claustrum (arrows, top row). Lesion locations are shown in red. Functional connectivity is displayed at a threshold of *T* < −7 (FWE *p* < 10^−6^)

In a final analysis, we examined functional connectivity between this brainstem cluster and the rest of the brain, focusing on anticorrelations. By definition, these anticorrelated regions are the areas that preferentially overlap LOC‐producing lesions (Figures 8 and S3). Peak anticorrelation with our brainstem node was identified in the bilateral claustrum, with additional peaks in the cingulate gyrus, bilateral temporal lobes, and lateral occipital cortices (Figure S4, Table S4).

## 4 DISCUSSION

In this study, we demonstrated a link between cortical lesions causing LOC and arousal centers in the dorsal brainstem. Cortical lesion locations were spatially heterogeneous. Analyses of lesion locations alone lacked sufficient power to detect any brain regions significantly associated with LOC. In contrast, connectivity between lesion locations and the dorsal brainstem was a strong and independent predictor of LOC. As such, connectivity with the dorsal brainstem defines a cortical network that, when lesioned, is most likely to cause LOC.

The current results are consistent with a growing literature showing that brain lesions causing similar symptoms localize to connected brain networks rather than individual brain regions (Fox, 2018). Lesions causing aphasia are connected to the left inferior frontal gyrus (Boes et al., 2015), lesions causing hemichorea are connected to the posterolateral putamen (Laganiere et al., 2016), and lesions causing freezing of gait are connected to the cerebellar locomotor region (Fasano et al., 2017). In this context, it is perhaps not surprising that lesions causing LOC are connected to the dorsal brainstem, the part of the brain known to be necessary for arousal and where lesions result in coma.

An important question is why we failed to find significant associations between lesion locations and LOC using traditional voxel lesion symptom mapping but did find significant associations using lesion network mapping. This may seem paradoxical given that both analyses are based on the same patients and lesion locations. The difference is that traditional lesion mapping looks for associations with individual brain regions, while lesion network mapping looks for associations with a distributed brain network (Fox, 2018). With limited sample sizes and heterogeneity in lesion location, only a small number of lesions will intersect a given brain region, but many more lesions will intersect a brain network. As such, the network approach increases the power for detecting significant associations, both in the current study and in prior studies (Ferguson et al., 2019; Padmanabhan et al., 2019).

That LOC‐producing lesions were *anticorrelated* rather than positively correlated to the dorsal brainstem was unexpected. This result was independent of connectome processing steps such as global signal regression, making it unlikely that this finding reflects a methodological artifact (Fox et al., 2005; Fox et al., 2009; Murphy & Fox, 2017). Anticorrelations, as seen with rs‐fcMRI, have been confirmed electrophysiologically (Keller et al., 2013), occur between regions modulated in opposite directions by tasks (Chai, Castanon, Ongur, & Whitfield‐Gabrieli, 2012; Fox et al., 2005), and appear to play an important role in linking lesion locations to lesion‐induced symptoms (Boes et al., 2015; Darby, Horn, Cushman, & Fox, 2018; Darby, Laganiere, et al., 2017). For example, lesions causing visual hallucinations are anticorrelated to extrastriate visual cortex (Boes et al., 2015), lesions causing auditory hallucinations are anticorrelated to auditory cortex (Boes et al., 2015), and both delusional misidentifications and criminality appear to depend in part on anticorrelations between the lesion location and other brain areas (Darby, Horn, Cushman, & Fox, 2018; Darby, Laganiere, et al., 2017). In the case of hallucinations, symptoms have been linked to hyperactivity in these anticorrelated brain regions (Boes et al., 2015). By extension, one might expect LOC to be associated with hyperactivity in the dorsal brainstem. Indeed, hyperactivity in the brainstem tegmentum has been reported during seizures producing LOC (Blumenfeld, 2012; Blumenfeld et al., 2009), in closed head injuries that result in LOC (Shaw, 2002), and in patients in a chronic vegetative state (Silva et al., 2010). Whether patients with lesion‐induced LOC show metabolic changes in the dorsal brainstem is a testable hypothesis for future work.

By definition, functional connectivity with this dorsal brainstem region defines a distributed brain network that encompasses lesion locations most likely to cause LOC (Figure 8). The peak of this network was in the superior claustrum, a region previously hypothesized to play an important role in human consciousness (Chau et al., 2015; Crick & Koch, 2005; Koubeissi, Bartolomei, Beltagy, & Picard, 2014). Prior work using this same lesion dataset found that lesions intersecting the claustrum were associated with the duration but not the frequency of LOC (Chau et al., 2015). The current study suggests that functional anticorrelation with the dorsal brainstem, rather than anatomical intersection with the claustrum, may be the more critical factor, explaining why many penetrating lesions outside the claustrum also cause LOC.

Previous work using anesthetic drugs has demonstrated that LOC is accompanied by alterations in anticorrelation between the default mode network and other networks including the executive control and salience networks (Bonhomme et al., 2016; Guldenmund et al., 2013). The set of brainstem‐anticorrelated regions identified here did not clearly fall within one of these canonical resting‐state networks (Yeo et al., 2011), but instead showed significant overlap with all three (Figure S8).

Our network aligns with some prior transcranial magnetic stimulation results. Stimulation of primary motor cortex and lateral (extrastriate) occipital cortex, both nodes in our network, has been associated with markers of impaired arousal. Stimulation of primary motor cortex induced delta waves on EEG, which are usually only seen in patients with impaired consciousness (Assenza, Pellegrino, Tombini, Di Pino, & Di Lazzaro, 2015), while stimulation of lateral occipital cortex, but not dorsolateral prefrontal cortex, showed effects on vigilance and arousal (Mensen, Gorban, Niklaus, Kuske, & Khatami, 2014).

While the lateral temporal and occipital cortices are not classically associated with LOC, accumulating evidence from lesion and functional neuroimaging suggests these regions, and the posterior brain in general, may play a role in conscious awareness (Boly et al., 2017). For example, traumatic lesions of the posterior corpus callosum are associated with an increased risk of permanent vegetative state (Kampfl et al., 1998) and posterior cortical lesions are associated with permanent coma after cardiac arrest (Bianchi & Sims, 2008). In our dataset, lesions intersecting lateral occipital nodes of our anticorrelated network tended to produce prolonged LOC, as indicated in Figure S3.

A lesion's propensity to cause LOC may be defined by a distributed brain network, with both anterior and posterior nodes. On the whole, our network appears to have a posterior predominance (Figures 8 and S4), consistent with some recent models of consciousness (Boly et al., 2017; Koch et al., 2016). However, it also includes some anterior regions, consistent with competing models (Dehaene & Naccache, 2001; Odegaard et al., 2017).

There are several limitations of the current study. First, this study measured connectivity between lesion location and other brain regions in a normative rs‐fcMRI database. This provides an estimate of each patient's own connectivity but cannot account for individual variation from the mean connectivity in a normal population. Second, determinations about LOC were made retrospectively. We cannot be certain whether all patients who appeared unconscious and did not follow commands truly lacked awareness. Furthermore, this dataset is inherently biased toward lesions that left no long‐term impairment on consciousness, since imaging was performed only on long‐term survivors (Salazar et al., 1986). Third, given the delay between injury and imaging it was not possible to determine extremely precise lesion boundaries. Fourth, unlike a recent study of brainstem infarctions (Fischer et al., 2016), many penetrating lesions were large and spanned multiple brain regions, which could impact our connectivity measurements. Fifth, symptoms from penetrating head trauma can result from factors not necessarily dependent on lesion location, like seizures or intracranial hypertension. Sixth, our lesions were based on CT scans acquired decades after the injury, which are insensitive to more subtle areas of damage or transient injury that may have contributed to LOC. Seventh, given that many years elapsed between injury and imaging, it is possible that certain areas of encephalomalacia arose from etiologies other than trauma. It is worth noting, however, that all of these limitations should bias us toward the null, against the positive findings in the current study. Finally, this study was performed in an exclusively male and predominantly white population, which limits its generalizability.

An interesting question is exactly which nuclei or cell types in the dorsal brainstem are likely to mediate lesion induced LOC. Answering this question in a definitive way is beyond the spatial resolution of imaging techniques such as fMRI, and is likely to benefit from ongoing work in animal models (Cho et al., 2017; Grandjean et al., 2019). This region of dorsal brainstem is composed of many different nuclei and neuronal subpopulations (Magoun, 1952; Verstegen, Vanderhorst, Gray, Zeidel, & Geerling, 2017; Wang & Morales, 2009). Some nuclei, like the locus coeruleus and parabrachial nucleus, primarily express excitatory neurotransmitters, promote arousal, and align well with the location of coma‐causing brainstem lesions (Fischer et al., 2016; Fuller et al., 2011; Magoun, 1952). Other neurons in this same area may actually suppress arousal or promote sleep, including interdigitating GABA and glycinergic neurons (Verstegen et al., 2017, Wang & Morales, 2009), as well as serotonergic neurons within the dorsal raphe (Denoyer, Sallanon, Kitahama, Aubert, & Jouvet, 1989; Sakai, 2011). This latter finding may be particularly relevant to the current results, as the peak connection associated with LOC overlapped the dorsal raphe.

Our findings implicating the dorsal raphe may have some support from animal studies. In a rat model, focal cortical lesions caused significant increases in raphe nuclei activity and serotonin release, which led to diffuse cortical hypometabolism (Tsuiki et al., 1995). Rat models of TBI have also confirmed diffuse extracellular serotonin increases immediately post injury (Busto, Dietrich, Globus, Alonso, & Ginsberg, 1997). Furthermore, optogenetic stimulation of the raphe nuclei in mice produced widespread reduction in cerebral blood volume (Grandjean et al., 2019). Finally, there are both excitatory and inhibitory reciprocal projections back to the raphe nuclei from cerebral cortex in rodents (Celada, Puig, Casanovas, Guillazo, & Artigas, 2001; Warden et al., 2012; Weissbourd et al., 2014), providing a possible anatomical substrate for these functional observations. Specifically, there are connections between the dorsal raphe and the claustrum (Pollak Dorocic et al., 2014), primary motor cortex (Beliveau et al., 2015; Pollak Dorocic et al., 2014), and extrastriate visual cortex (Tigges, Walker, & Tigges, 1983). Motivated by these convergent findings, a testable model of LOC induced by cortical lesions is released inhibition on serotonergic nuclei in the brainstem, which in turn exert diffuse suppressive effects on the cortex, either directly or through effects on adjacent ARAS nuclei (Grandjean et al., 2019; Tsuiki et al., 1995).

Herein, we offer the first connection between heterogeneous, LOC‐producing cortical lesions and the brainstem. Future work is needed to clarify the functional relationship between specific brainstem nuclei and nodes of this cortical network.

## CONFLICT OF INTERESTS

S.B.S., J.H., R.R.D., D.C., D.B.F., A.L.C., and J.H.G. report no relevant disclosures. M.D.F. is an author on submitted patents using brain connectivity to understand and treat neuropsychiatric symptoms.

## Supporting information


**Appendix S1.** Supporting Information

## Data Availability

The data that support the findings of this study are available from J.H.G. upon reasonable request and approval of the Northwestern University IRB.
