# Supplementary material for: Cortical lesions causing loss of consciousness are anticorrelated with the dorsal brainstem
Source: Hum Brain Mapp. 2020 Jan 6;41(6):1520–31. doi: 10.1002/hbm.24892 (PMC7268053; doi:10.1002/hbm.24892)
Supplement: Supplementary file 1 — Appendix S1. Supporting Information [file HBM-41-1520-s001.pdf]

Supplementary Information for

Cortical lesions causing loss of consciousness are anticorrelated with the dorsal brainstem

Samuel B. Snider, MD, Joey Hsu, R. Ryan Darby, MD, Danielle Cooke, David Fischer, MD, Alexander L. Cohen, MD, PhD, Jordan H. Grafman, PhD, Michael D. Fox, MD, PhD

Corresponding: Samuel B. Snider, MD
Email: ssnider@partners.org

This PDF file includes:

- Supplementary text
- Tables S1-4
- Figures: S1-8
- References for SI reference citations

Supplementary Information Text

Supplementary Methods

Lesion characteristics

Lesion Volume:

Defined as total number of (2mm isotropic) voxels in the binary mask in MNI152 space.

Left/right hemispheric:

These were defined as lesions having at least 20 lesioned voxels on the respective side of the mid-sagittal plane in MNI space. If lesions had more than 0 but fewer than 30 voxels on a given side, they were visually inspected to confirm hemispheric involvement.

Bilateral:

We sought to capture all lesions with parenchymal supratentorial involvement in each hemisphere. We defined these lesions as having >10 voxels lesioned in each hemisphere on either side of an 8-voxel window spanning the midline. Lesions with more than 0 but fewer than 10 voxels were visually inspected for confirmation.

Posterior-fossa:

Due to the relatively small number of lesions with posterior fossa involvement, we first identified the subset of all lesions with even one voxel involving the brainstem or cerebellum, via intersection with the Harvard-Oxford subcortical atlas mask (threshold

0). Lesions had to involve at least 10 cerebellar voxels or at least 5 brainstem voxels to be classified as having cerebellar or brainstem involvement respectively.

Thalamic:

Due to the small number of thalamic lesions, we first identified lesions with at least one voxel overlapping the thalamus (Harvard-Oxford subcortical atlas mask, threshold 0). We then excluded several lesions that were primarily cortical but had deep extension and fewer than 5 voxels of peripheral thalamic involvement. All our lesions classified as having thalamic involvement had more than 10 voxels lesioned within this thalamic mask and qualitatively showed unequivocal thalamic involvement. There were two lesions with more than 10 lesioned voxels but qualitatively peripheral thalamic involvement. Excluding these two lesions had no effect on analyses requiring identification of thalamic lesions.

A-priori region of interest

Coma-specific area:

This area was identified from a VLSM analysis of 12 cases of acute brainstem lesions producing coma and 24 acute brainstem lesions without effect on level of consciousness. As described previously(Fischer, Boes et al. 2016), a Z score was computed for each voxel, representing the likelihood that a lesion interesting the voxel would produce coma. To generate our ROI, this map was thresholded at $Z \geq 3.54$, which led to FDR-corrected $P < 0.05$. The resulting ROI was used as a weighted-seed in subsequent functional connectivity analyses.

Lesion Analyses

Lesion characteristics

Each lesion characteristic (lesion volume, brainstem involvement, thalamic involvement, posterior fossa involvement, left/right hemisphere involved, bilaterality) was tested for association with LOC in an ordinal logistic regression using a proportional odds model. For no predictor variable was the proportional odds assumption violated (Likelihood Ratio Test of non-proportional odds, P > .2). The model including volume and hemisphere had a better fit than a model containing volume or hemisphere alone (LR 9.3, P = .002) but was not significantly affected by the addition of bilaterality (LR 2.1, P = .1).

Voxel-wise

We performed voxel-wise regressions between the presence of a lesion at each individual parenchymal voxel and LOC, defined ordinally. We restricted the analysis to voxels lesioned at least five patients (Figure S5). VLSM studies require such thresholds to avoid *misplacement* biases(Karnath, Sperber et al. 2017). Because there is no clear theoretical reason require a five-patient minimum, we repeated the analysis with a ten-patient threshold. In both cases, controlling the FDR (q = .05) using Benjamini-Hochberg procedure(Benjamini 1995), we found no significant voxels. We also found no significant voxels when treating LOC as a binary variable and using voxel-wise Chi-squared or Fisher exact tests.

Lesion Network Mapping

ROI-to-ROI

Lesions' connectivity with coma-specific area (ROI to ROI analyses)

The addition of lesion connectivity to the coma-specific area improved a model including lesion volume (LR 6.7, $P < .01$), or lesion volume and hemisphere (LR 11.2, $P < .001$). To confirm our results were not biased by deep lesions, we excluded the 20 lesions with involvement of the thalamus, brainstem, or cerebellum and repeated the regressions, with unchanged results. After exclusion of these lesions, connectivity to the brainstem ROI remained significantly associated with LOC ($P = 0.01$ univariate, $P = 0.009$ multivariate with lesion volume, and $P = 0.0008$ multivariate with lesion volume and left-hemispheric involvement).

Voxel-wise

For the voxel-wise analyses, we generated, for each lesion, the connectivity (Fisher Z transformed Pearson's R) between the binary lesion mask and every voxel within a midbrain and pons-restricted mask, automatically segmented from the MNI T1 2mm brain using Freesurfer. We first used FSL's PALM tool to run voxel-wise GLMs using Fz values to predict LOC defined ordinally, separately including lesion size and left-hemispheric involvement in the models. Significant voxels were FWE-corrected $P < 0.05$ using PALM's threshold free cluster enhancement.

To find the whole-brain peaks, we performed logistic regressions at every voxel within a whole brain T1 2mm MNI brain mask, again including the above covariates in each regression. We used a raw P value threshold of 0.001 to identify the peaks (Figure S1).

Control Analyses

Anti-correlated network

Voxels with FWE corrected P < 0.05 in all voxel-wise analyses using PALM (no covariates, lesion volume, or lesion volume and hemisphere) were used to create a seed region. We computed the Fisher Z-transformed Pearson R between this seed region and every other voxel within the MNI brain mask. The anticorrelated regions represent the areas preferentially intersected by LOC-causing lesions (Figure S4). We used FSL's cluster function to define the coordinates of the peaks (cluster size > 50 voxels, T value > 7) (Table S4).

Given the proximity of the seed to the 4th ventricle, we sought to ensure our anti-correlated network map was not driven by CSF signal. We created a spherical ROI entirely within the 4th ventricle and subsequently computed the partial correlation between our seed and every other voxel in the brain mask, controlling for the correlation between the 4th ventricular seed and every other voxel (Figure S6). The majority of the peaks from the original anticorrelated network, with the exception of M1 and the left cingulate, remained significant.

Brainstem temporal signal-to-noise ratio (tSNR)

Given that our connectivity findings mapped to the brainstem tegmentum, we further investigated the temporal signal to noise ratio (tSNR) within this region of brainstem to ensure the presence of reliable BOLD signal in this region.

We calculated the voxel-wise mean tSNR from the Brain Genomics Superstruct Project set of N = 1570 normative rs-fMRI scans (Holmes, Hollinshead et al. 2015) used in the analyses in the manuscript. fMRI data from each subject was processed through fmriprep (https://www.ncbi.nlm.nih.gov/pubmed/30532080), applying standard fMRI preprocessing steps as described in the cited methods. Voxel-wise tSNR (mean of

BOLD time series / standard deviation of BOLD time series) was then calculated for each subject using a python script: fmri_tsnr.py available here: (https://gist.github.com/alexlicohen/). The group average tSNR for the entire brain and for the pons-midbrain mask utilized in the manuscript were calculated using FSL (http://www.ncbi.nlm.nih.gov/pubmed/21979382) command line tools.

Representative slices for the whole-brain and brainstem voxel-wise tSNR are shown in Figure S7. Brainstem tSNR was examined within the freesurfer-generated mask of the pons and midbrain used in the manuscript. Mean tSNR was indeed lower *inside* (mean 51.1, CI: (30.7,71.5)) than *outside* (72.2 (32.2,112.2)) the brainstem mask, (CI for difference: -20.7, -21.5, T = -111.2, p < 0.0001). However, tSNRs > 50 are relatively robust and prior reports have suggested that tSNR > 20 provides consistent and reproducible functional connectivity results (Barry, Conrad et al. 2018).

To determine whether voxel-wise tSNR differences biased the topography of our brainstem cluster, we next examined whether brainstem tSNR differed inside versus outside of this cluster (Figure S7-B,C). There was no difference in median brainstem tSNR *inside* (53.6, IQR (47.7,56.6)) as compared with *outside* 53.4 (44.0,58.9) this cluster (Z = -0.3, p = 0.8). This equivalency, along with the uniform appearing map of brainstem tSNR (Figure S7-B), argues that the location of our cluster was not biased by signal inhomogeneity within the brainstem.

Lesion and network intersection with canonical resting-state networks.

Previous work has demonstrated that sedating drugs that cause impairment of consciousness alter the degree of anticorrelation between the default mode network and other brain regions (Guldenmund, Demertzi et al. 2013, Bonhomme, Vanhaudenhuyse

et al. 2016). We sought to investigate whether the network of regions anti-correlated to the brainstem cluster preferentially intersected one of three canonical rs-fMRI networks felt to play a role in conscious information processing: the default mode network, the executive control network and the salience network. For this analysis, we used the 7-network resting state cortical parcellation in MNI space (https://surfer.nmr.mgh.harvard.edu/fswiki/CorticalParcellation_Yeo2011) (Yeo, Krienen et al. 2011). We found that the un-thresholded network of regions anticorrelated to the brainstem cluster overlapped all three networks, with a slight preference for the DMN and Salience Networks (Figure S8).

Temporal and Occipital Network Nodes

We sought to investigate the relevance of our temporal and occipital anticorrelated network nodes specifically in contributing to lesions' association with LOC. We used the Harvard-Oxford atlas labels of the temporal and occipital cortical regions in MNI space (https://fsl.fmrib.ox.ac.uk/fsl/fslwiki/Atlases), thresholded at 50 at binarized. Restricting our analysis to the N = 92 lesions in our dataset intersecting these temporal and occipital cortical masks, connectivity with the coma-specific area remained a significant predictor of LOC (B = 1.2 {per -.01 change in Fz}, p = 0.03). This is illustrated with representative lesions in Figure S3.

Table S1: Baseline patient characteristics

	Full Cohort (N = 171)	No LOC (N = 64)	Brief LOC (N = 91)	Prolonged LOC (N = 16)	Chi-Sq, (P value)
**Age at 35-year follow-up (yrs), Median (IQR)**	58 (57,59)	57 (56,59)	58 (57,60)	58 (57,59)	2.4 (0.3)
**Education (yrs), Median (IQR)**	14 (12,16)	14 (13,16)	15 (12,17)	14 (12,16)	1.6 (0.5)
**Handedness (R, %)**	144 (84%)	55 (86%)	76 (84%)	13 (81%)	0.5 (0.9)
**Race (White, %)**	154 (90%)	58 (91%)	80 (88%)	16 (100%)	2.4 (0.5)
**Ethnicity (non-Hispanic)**	165 (96%)	62 (97%)	87 (96%)	16 (100%)	0.9 (0.8)

Table S2: Lesion characteristics associated with LOC.

	**# of lesions (total 171)**	***B***	**Univariate *p***
**Lesion volume**	171	1.000084*	.00011
**Left hemispheric**	95	3.07	.00033
Right hemispheric	116	1.02	.95
Posterior fossa	13	.59	.33
**Bi-hemispheric**	41	3.71	.00059

*per *voxel* of lesion volume

Table S3: Lesion characteristics independently associated with LOC in a multivariate regression

	# of lesions (total 171)	*B*	P
**Lesion volume**	**171**	**1.000069**	**.0023**
**Left hemispheric**	**95**	**2.10**	**.033**
Bi-hemispheric	41	1.87	.15

Table S4. MNI coordinates of anti-correlated network peaks*

**ROI**	**MNI coordinates (mm)**	**Peak (T) Value**
Claustrum (L)	-36, -6, -12	13.5
Claustrum (R)	36 , -2 , 10	13.4
L temporal tip	-56, 14, -8	12.6
Cingulate (R)	10, -14, 38	12.3
Amygdala (L)	-20, 0 , -18	12
Inferior temporal (L)	-42, -14, -34	10.7
Amygdala (R)	22, 0 , -18	10.2
M1 (L) *A*	-36, -42, 68	10.1
L medial frontal	-6,-10,76	9.79
M1 (L) *B*	-48, -10, 54	9.66
Cistern	2, -32, 2	9.85
Inferior temporal (R)	50, -38, -18	9.4
Lat. Occipital (R)	34, -82, -14	8.73
R temporal	52, -18, -6	8.27
M1 (R)	46, -34, 64	8.18

* MNI T1 2mm brain-masked, threshold T > 7, cluster size > 50 voxels

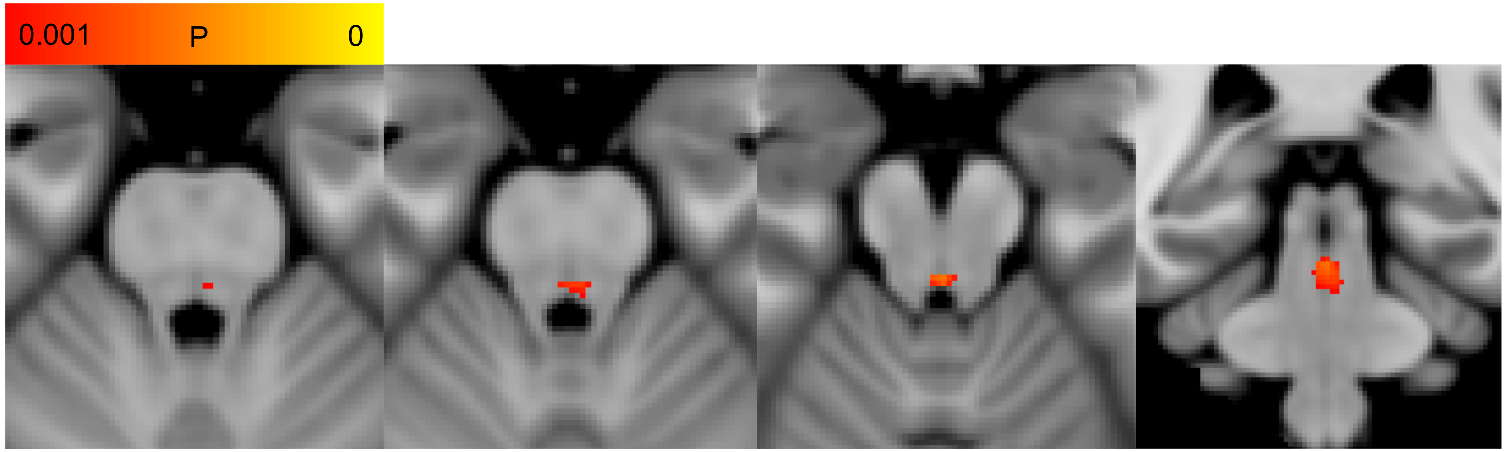

Figure S1. Whole brain peak-search

Voxel-wise correlations between lesions' connectivity and LOC, in multivariate regressions including lesion volume and left-hemisphere, within a T1 2mm brain mask. At a raw P threshold of 0.001, the whole-brain grey matter peak is shown.

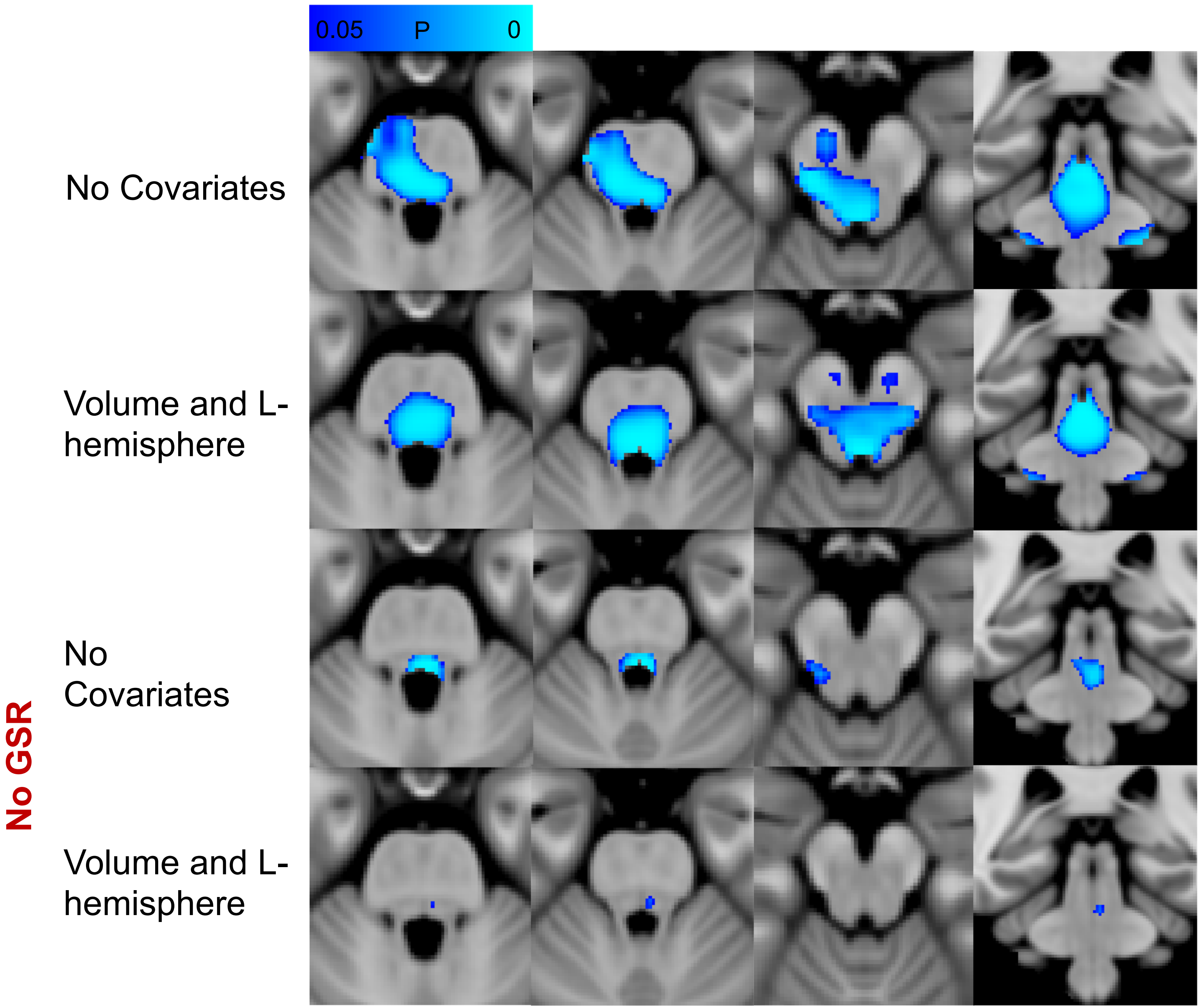

Figure S2. Comparison of brainstem peaks

Four axial brainstem slices are shown demonstrating the area overlapped by the brainstem cluster to which lesion connectivity predicted LOC (blue voxels represent P < 0.05). The same area is overlapped with or without covariate inclusion, and when repeated on a subset (N = 100) normative connectomes processed without use of the global signal regression.

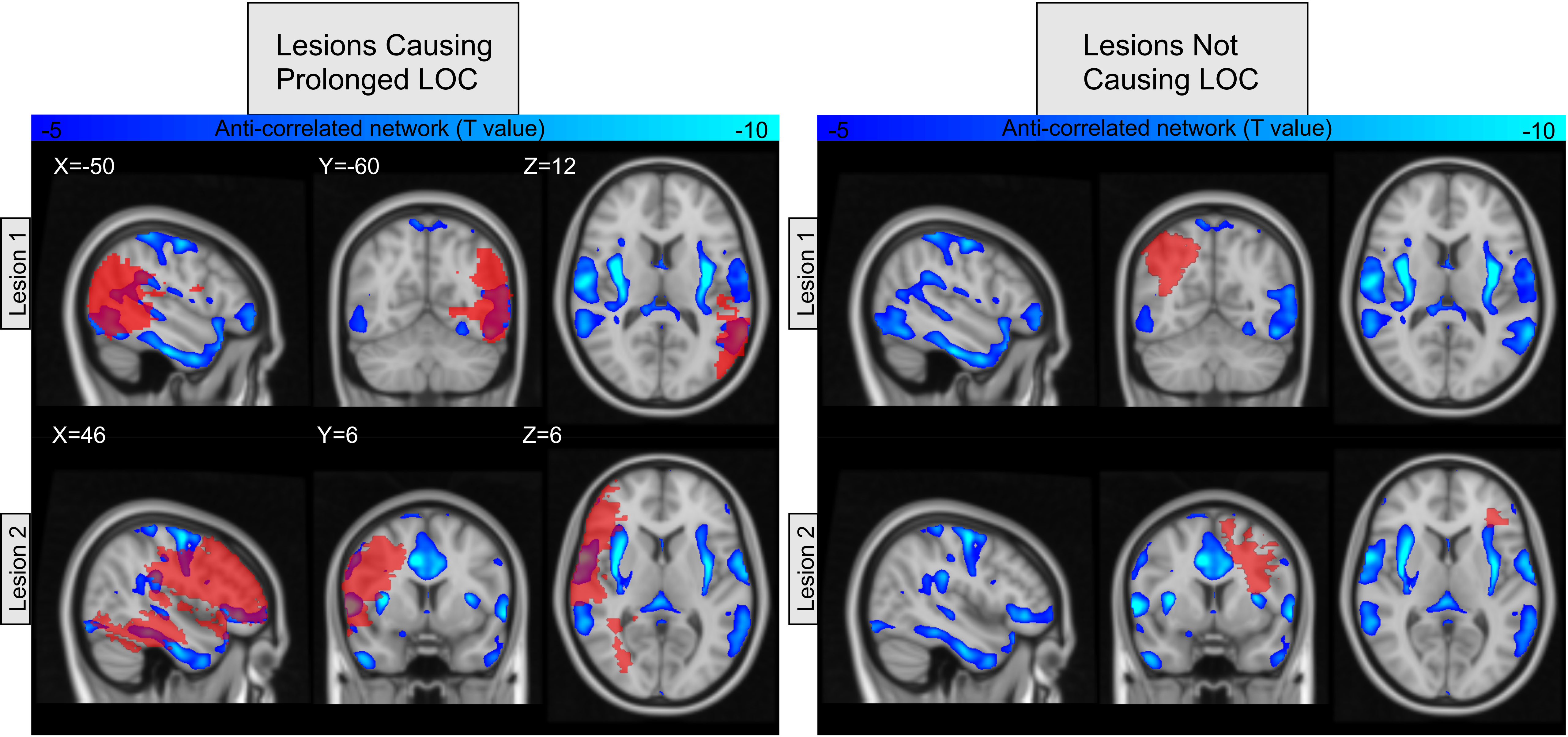

Figure S3: Lesion overlap with brainstem anti-correlated network.
3 views (sagittal, coronal and axial) of two representative lesions causing prolonged (> 1 day) LOC compared with two lesions that did not cause LOC. The lesions are indicated in red and the anti-correlated network is shown in blue. Lesions causing prolonged LOC have extensive overlap with lateral occipital and temporal network nodes, while lesions not causing LOC spare these regions.

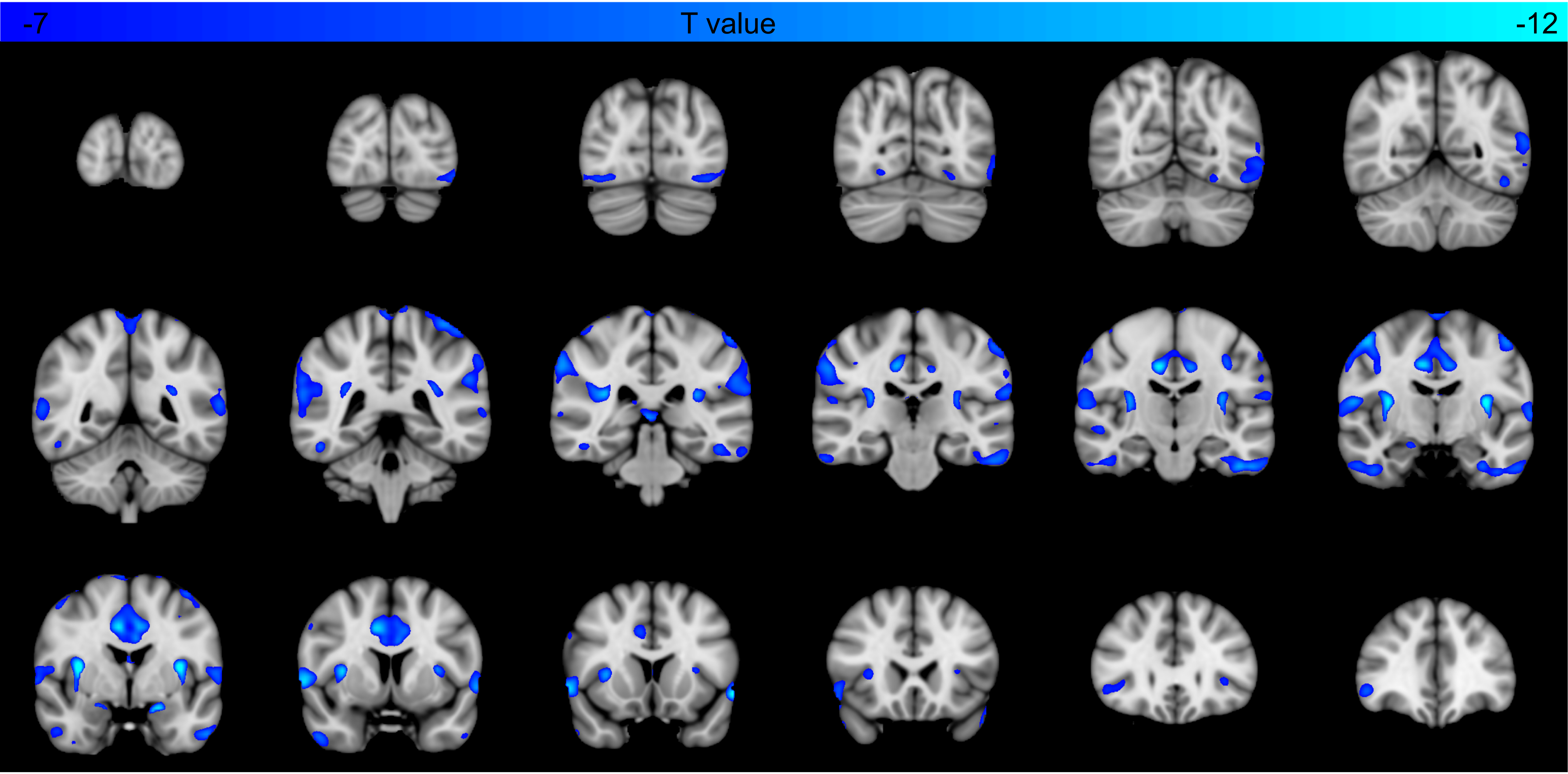

Figure S4. Anticorrelated network

Coronal slices of the network of brain regions anticorrelated ($T \leq -7$) to the peak brainstem cluster (Figure 5B).

Voxels lesioned in ≥ 5 patients

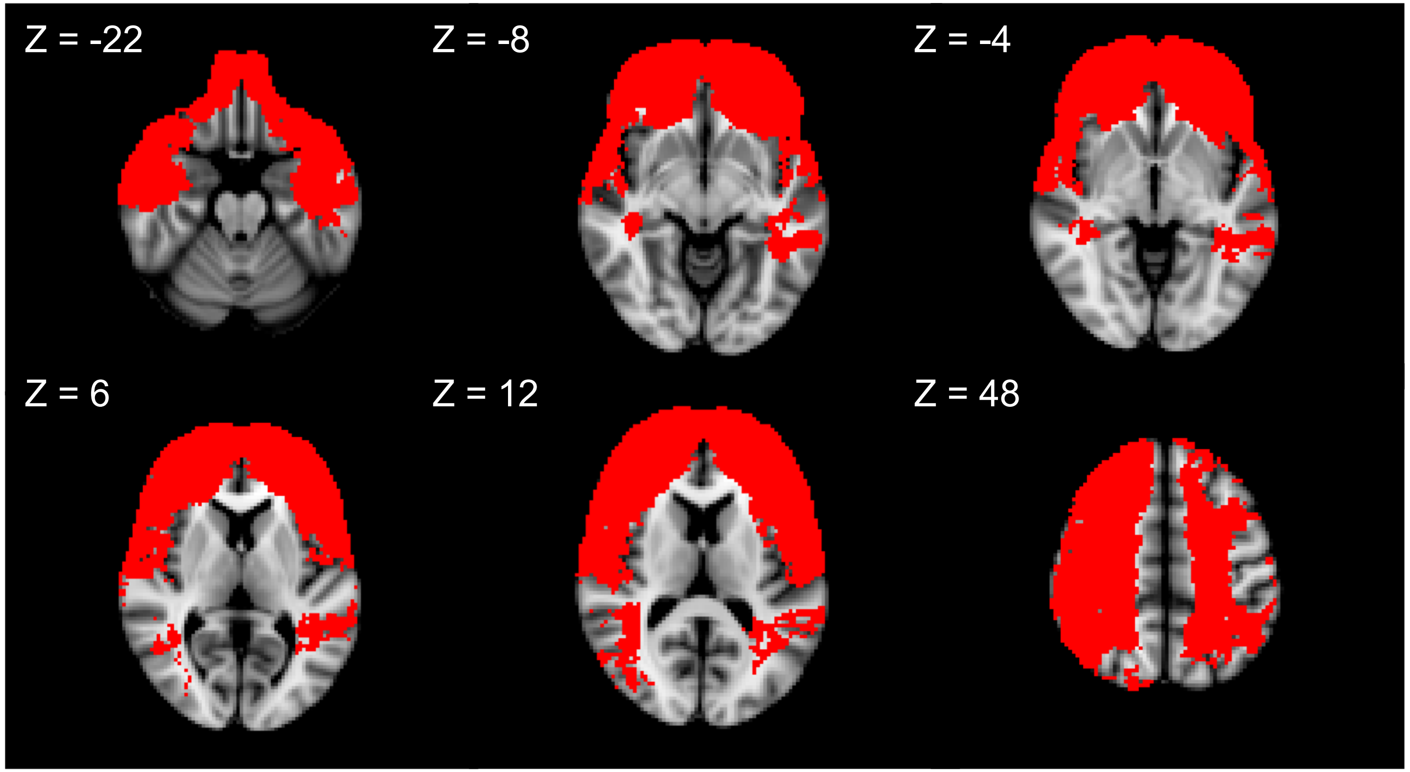

Figure S5. VLSM coverage

Map of voxels lesioned in more than five patients are shown, representing our overall coverage for the initial VLSM analyses. There are notable vacancies over the claustrum and occipital cortices, peaks of the brainstem anticorrelated network, offering a potential explanation for the negative VLSM results in this study.

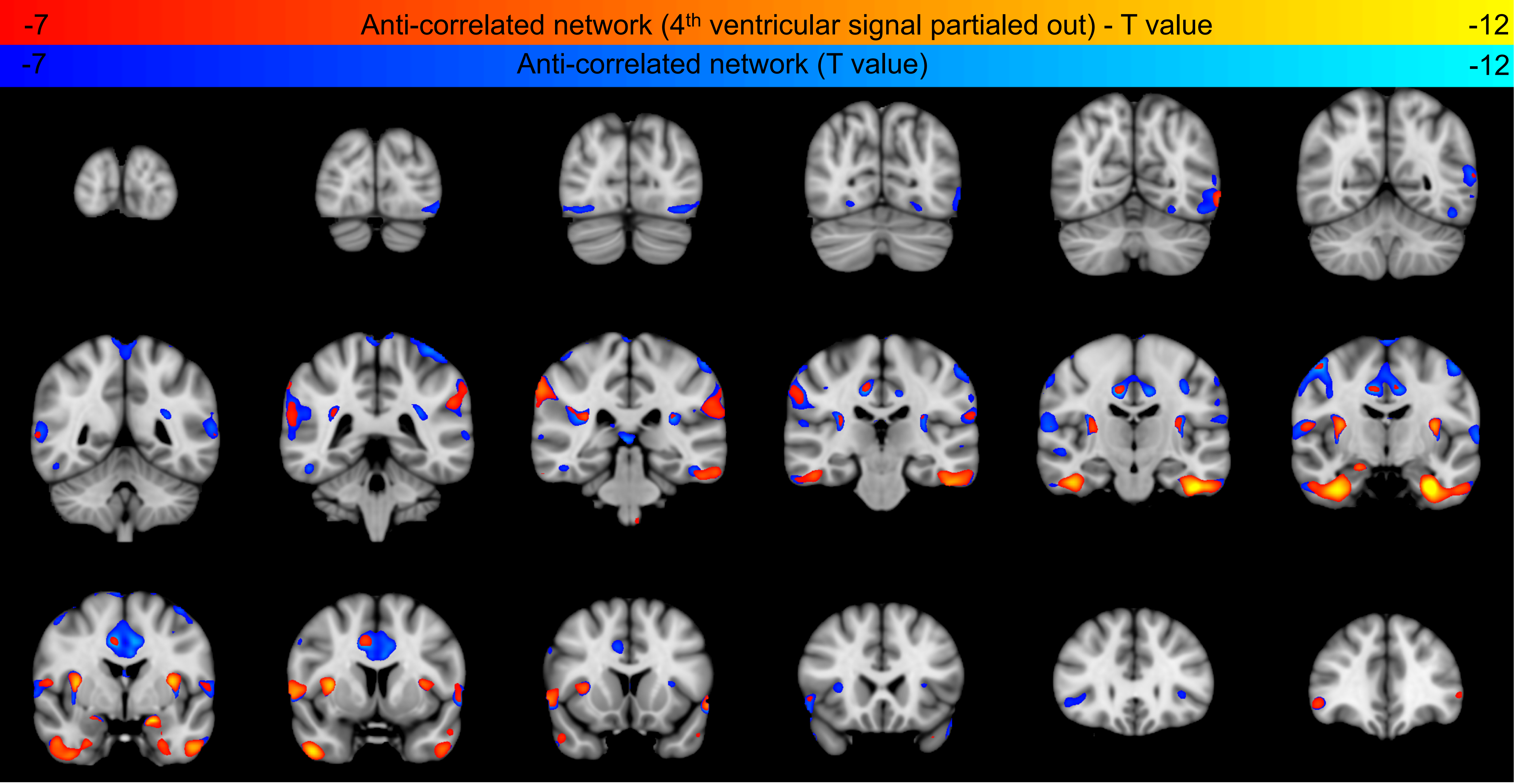

Figure S6. Anti-correlated network, 4th ventricular signal removed Coronal slices showing the resulting network anticorrelated ($T \leq -7$) to the brainstem peak (Figure 5B) after controlling for connectivity to the 4th ventricle (CSF signal). The left cingulate, M1, and lateral occipital regions are diminished in significance, but the network is otherwise similar.

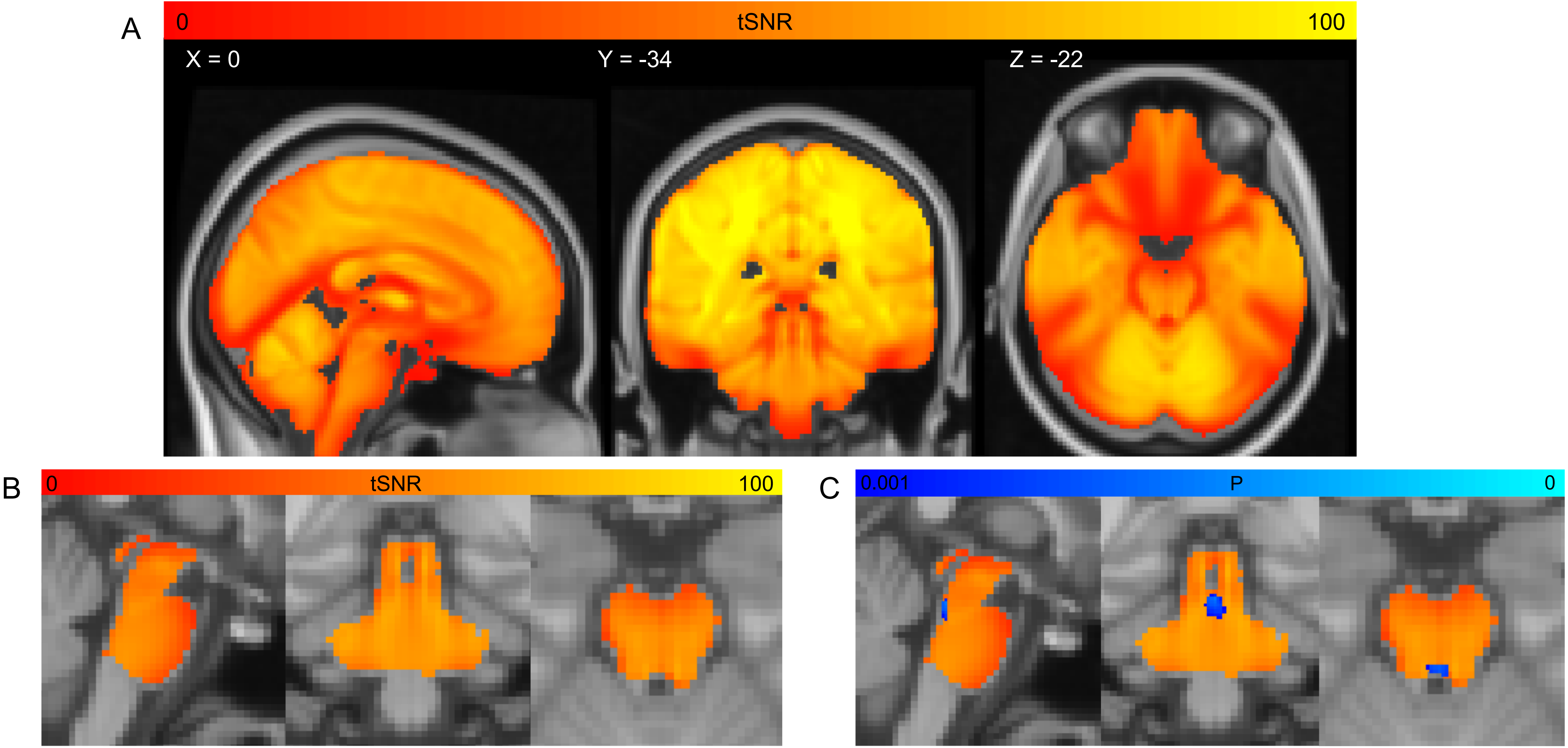

Figure S7: Voxel-wise tSNR
(A) Voxel-wise mean temporal signal to noise ratios (tSNRs) from the Brain Genome Superstruct Project set of N = 1570 rs-fMRI scans are plotted in MNI space at the indicated sagittal, coronal and axial slices. (B) Zoomed-in voxel-wise tSNR within an automated freesurfer segmentation of the pons and midbrain midbrain at the above slices. (C) Peak brainstem cluster associated with LOC (raw p threshold < 0.001) plotted on this map.

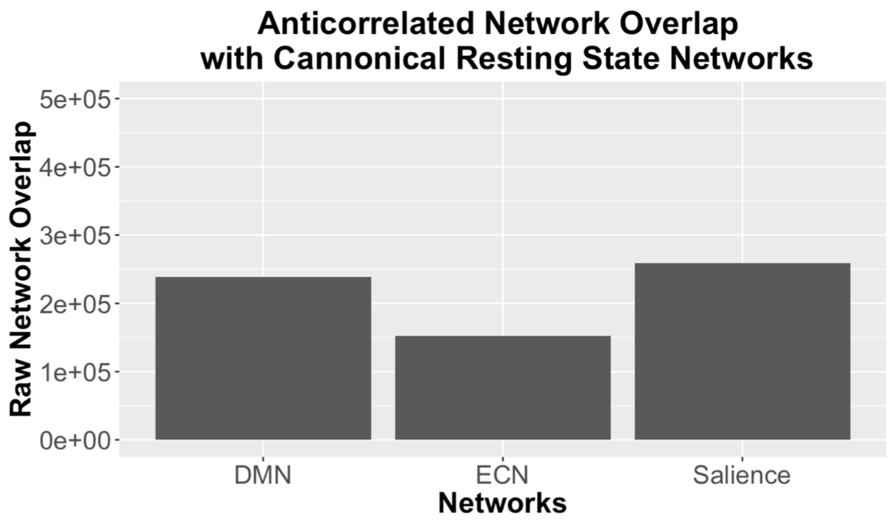

Figure S8: Anti-correlated network overlap with Default Mode (DMN), Executive Control (ECN) and Salience Networks.
Raw summed T value overlap of the three canonical networks with the network of regions anti-correlated to the brainstem tegmentum cluster from Figure 5 (Main text).

References

Barry, R. L., B. N. Conrad, S. A. Smith and J. C. Gore (2018). "A practical protocol for measurements of spinal cord functional connectivity." Sci Rep **8**(1): 16512.

Benjamini, Y. H., Yosef (1995). "Controlling the false discovery rate: a practical and powerful approach to multiple testing." Journal of the Royal Statistical Society. Series B **57**(1): 289-300.

Bonhomme, V., A. Vanhaudenhuyse, A. Demertzi, M. A. Bruno, O. Jaquet, M. A. Bahri, A. Plenevaux, M. Boly, P. Boveroux, A. Soddu, J. F. Brichant, P. Maquet and S. Laureys (2016). "Resting-state Network-specific Breakdown of Functional Connectivity during Ketamine Alteration of Consciousness in Volunteers." Anesthesiology **125**(5): 873-888.

Fischer, D. B., A. D. Boes, A. Demertzi, H. C. Evrard, S. Laureys, B. L. Edlow, H. Liu, C. B. Saper, A. Pascual-Leone, M. D. Fox and J. C. Geerling (2016). "A human brain network derived from coma-causing brainstem lesions." Neurology **87**(23): 2427-2434.

Guldenmund, P., A. Demertzi, P. Boveroux, M. Boly, A. Vanhaudenhuyse, M. A. Bruno, O. Gosseries, Q. Noirhomme, J. F. Brichant, V. Bonhomme, S. Laureys and A. Soddu (2013). "Thalamus, brainstem and salience network connectivity changes during propofol-induced sedation and unconsciousness." Brain Connect **3**(3): 273-285.

Holmes, A. J., M. O. Hollinshead, T. M. O'Keefe, V. I. Petrov, G. R. Fariello, L. L. Wald, B. Fischl, B. R. Rosen, R. W. Mair, J. L. Roffman, J. W. Smoller and R. L. Buckner (2015). "Brain Genomics Superstruct Project initial data release with structural, functional, and behavioral measures." Sci Data **2**: 150031.

Karnath, H. O., C. Sperber and C. Rorden (2017). "Mapping human brain lesions and their functional consequences." Neuroimage **165**: 180-189.

Yeo, B. T., F. M. Krienen, J. Sepulcre, M. R. Sabuncu, D. Lashkari, M. Hollinshead, J. L. Roffman, J. W. Smoller, L. Zollei, J. R. Polimeni, B. Fischl, H. Liu and R. L. Buckner (2011). "The organization of the human cerebral cortex estimated by intrinsic functional connectivity." J Neurophysiol **106**(3): 1125-1165.
